# PD-L1-Expressing Dendritic Cells Contribute to Viral Resistance during Acute HSV-1 Infection

**DOI:** 10.1155/2012/924619

**Published:** 2012-02-28

**Authors:** Katie M. Bryant-Hudson, Daniel J. J. Carr

**Affiliations:** Department of Ophthalmology, Dean McGee Eye Institute, The University of Oklahoma Health Sciences Center, Room 415, 608 Stanton L Young Boulevard, Oklahoma, OK 73104, USA

## Abstract

The inhibitory receptor, Programmed Death 1 (PD-1), and its ligands (PD-L1/PD-L2) are thought to play a role in immune surveillance during chronic viral infection. The contribution of the receptor/ligand pair during an acute infection is less understood. To determine the role of PD-L1 and PD-L2 during acute ocular herpes simplex virus type 1 (HSV-1) infection, HSV-1-infected mice administered neutralizing antibody to PD-L1 or PD-L2 were assessed for viral burden and host cellular immune responses. Virus titers were elevated in cornea and trigeminal ganglia (TG) of anti-PD-L1-treated mice which corresponded with a reduced number of CD80-expressing dendritic cells, PD-L1^+^ dendritic cells, and HSV-1-specific CD8^+^ T cells within the draining (mandibular) lymph node (MLN). In contrast, anti-PD-L2 treatment had no effect on viral replication or changes in the MLN population. Notably, analysis of CD11c-enriched MLN cells from anti-PD-L1-treated mice revealed impaired functional capabilities. These studies indicate PD-L1-expressing dendritic cells are important for antiviral defense during acute HSV-1 infection.

## 1. Introduction

The inflammatory response to microbial pathogens can have detrimental consequences to the host especially at vulnerable sites such as the eye. Fungal, bacterial, and viral infections within the anterior segment of the eye can lead to significant infiltration of leukocytes as well as angiogenesis (both lymph- and hemangiogenesis) in the cornea [1, 2]. Herpes simplex virus type 1 (HSV-1) is a neurotropic member of the alpha herpes virus family and a common human pathogen that infects 60–90% of the adult worldwide population [3]. An HSV-1 infection can have devastating consequences to vision as a result of a robust immune response to episodic reactivation of latent virus from reservoirs found in the sensory ganglion (i.e., trigeminal ganglion [TG]) [4]. Reactivation begins with the resumption of the lytic viral replication cycle in infected neurons. Infectious virions then travel down trigeminal nerve fibers to epithelial surfaces via anterograde axonal transport. The trigeminal nerve provides sensation to the lips, nose, and eye; therefore, each site is susceptible to infection following reactivation.

Reactivation of latent HSV-1 results in repeated inflammation and scarring in the stromal layer of the cornea which can eventually progress to herpetic stromal keratitis (HSK) [1, 5]. While there are a number of leukocyte subpopulations that contribute to tissue pathology, CD4^+^ Th1 cells play a key role with the production of interferon-*γ* (IFN-*γ*) [1, 5]. Consequently, control of the inflammatory nature of the immune response to HSV-1 is a desirable outcome in order to preserve the visual axis.

There are a number of candidate molecules and pathways that have been shown to contribute to tissue pathology. One such pathway is the PD-1 : PD-L signaling pathway, a negative regulatory pathway that results in T-cell exhaustion. Programmed Death 1 (PD-1) is a cell surface inhibitory receptor found on activated T cells. Programmed Death Ligand 1 (PD-L1) is found on resting T cells, B cells, macrophages, and dendritic cells, whereas expression of Programmed Death Ligand 2 (PD-L2) is restricted to macrophages, dendritic cells, and activated T cells [6–8]. T-cell exhaustion includes a loss of IL-2 production and a decrease in proliferation and killing capabilities [9]. PD-1:PD-L signaling also plays an important role in the maintenance of peripheral cell tolerance and the generation of induced regulatory T cells [10, 11]. PD-1 : PD-L signaling also has been extensively studied in the context of chronic viral infections such as LCMV, HIV, and HCV [12–14]. During LCMV or HIV infections, reduction in T-cell exhaustion via blockade of PD-1 : PD-L signaling significantly reduces the viral load [12, 13]. During chronic viral infection many factors can contribute to T-cell exhaustion including the level of antigen exposure, availability of CD4 T-cell help, and changes in antigen presenting cell (APC) numbers [13]. Moreover, changes in costimulatory molecule expression on the surface of APCs can dramatically alter T-cell effector function and the course of viral infection [14].

Following ocular infection with HSV-1, PD-1 expression is upregulated on CD4 T cells in both the cornea and draining lymph node [15]. Similarly, PD-L1 is up-regulated on macrophages in both tissues [15]. Studies have also indicated blocking PD-1 : PD-L signaling during HSV-1 enhances pathology by increasing the proliferation of HSV-specific CD4 T cells that secrete IFN-*γ* [15]. Recent studies have indicated a correlation between the levels of latent HSV-1 and the expression of PD-1 [16, 17]. However, no studies have evaluated the impact of PD-1 : PD-L signaling during acute HSV-1 infection. To address this issue we compared HSV-1-infected mice administered neutralizing antibody to PD-L1 and PD-L2 in terms of viral replication in infected tissues, the host cellular immune response phenotypically and functionally within the cornea, TG, and draining lymph node, and characterization of select intracellular signaling molecules central to T-cell activation. Results from this study indicate PD-L1 has a unique role during HSV-1 infection, wherein blockade of PD-1 : PD-L1 signaling decreases the activation of dendritic cells resulting in an increased viral load.

## 2. Materials and Methods

### 2.1. Virus and Mice

C57BL/6J mice were obtained from The Jackson Laboratory and maintained at Dean McGee Eye Institute. HSV glycoprotein-B- (gB-) specific T-cell receptor transgenic mice were obtained from Dr. Francis Carbone (University of Melbourne) and maintained at Dean McGee Eye Institute. Animal treatment was consistent with the National Institutes of Health Guidelines on the Care And Use of Laboratory Animals. All procedures were approved by the University of Oklahoma Health Sciences Center and Dean McGee Eye Institute Institutional Animal and Care Use Committee. HSV-1 (strain McKrae) was grown and maintained as previously described [18].

### 2.2. HSV-1 Infection and Neutralizing Antibody Treatment

Male and female C57BL/6 mice (6–10 wk of age) were anesthetized by intraperitoneally (i.p.) injection with xylazine (6.6 mg/kg) and ketamine (100 mg/kg) followed by scarification of the cornea using a 25 5/8-guage needle. The tear film was then blotted, and the cornea was topically inoculated with 1,000 plaque forming units (PFU) of HSV-1 in 3 *μ*L of RPMI-1640 medium. At days 2, 4, and 6 after infection (p.i.) mice were administered 200 *μ*g of neutralizing antibody to PD-1, PD-L1, PD-L2, or isotype control (IgG) by i.p. injection. Neutralizing antibodies and Rat IgG were obtained from BioXcell. HSV-1 viral titers were determined in the designated tissue at times p.i. by plaque assay as previously described [19].

### 2.3. Flow Cytometry

At the indicated time p.i., mice were exsanguinated and the cornea, draining (mandibular) lymph node (MLN), and TG were removed, processed, labeled with Abs, and analyzed using a Coulter Epics XL flow cytometer (Beckman coulter) with the absolute number of cells residing in the indicated tissue determined as previously described [20]. The following Abs were used for the identification of cell populations; anti-CD3 (1742), anti-CD4 (RM4-5), anti-CD8*α* (53-6.7), anti-NK1.1 (PK136), anti-CD45 (30-F11), anti-F4/80 (MCA497FA), anti-GR1 (RB6-8C5), anti-CD11c (HL3), anti-B220 (RA3-6B2). For tetramer staining, cells were labeled with HSV peptide gB_498–505_ (SSIEFARL)-specific major histocompatibility complex tetramer (MHC Tetramer Lab, Baylor College of Medicine), anti-CD8, and anti-CD45. Single cell suspensions of MLN and cornea samples were also evaluated for T_reg_ cells using a commercial kit (eBiosciences).

### 2.4. Suspension Array

At the indicated time p.i., cornea, TG, and MLN were removed from the exsanguinated mice and assayed for the detection of CXCL1, CCL2, CCL5, and IFN-*γ* using a suspension array system (Bio-Rad).

### 2.5. ELISA

At the indicated time p.i., the TG and cornea were removed from the exsanguinated mice and placed in 500 *μ*L of 1X PBS containing a protease inhibitor mixture (Calbiochem) on ice. Following homogenization with a tissue miser (Fisher Scientific), the homogenates were clarified by centrifugation at 10,000 × g for 1 min. CXCL10 levels were determined by ELISA according to the manufacturer's instructions (Quantikine immunoassay; R and D Systems).

### 2.6. MLN T-Cell Cocultures

To assess MLN T-cell function of HSV-1-infected mice treated with anti-PD-L1, anti-PD-L2, or control IgG, CD11c^+^ cells were purified from the spleen of untreated-C57BL/6 mice using MACS beads and column (Miltenyi Biotec). One hundred thousand CD11c-enriched cells were seeded into a 96-well round-bottom plate and pulsed with UV-inactivated HSV-1 at a multiplicity of infection (MOI) of 5 for 2 hrs. Following incubation, the media was removed from the CD11c-enriched cells and single-cell suspensions of 1 × 10^6^ MLN cells from HSV-1 infected mice treated with anti-PD-L1, anti-PD-L2, or control IgG were added. Cultures were incubated for 6 days at 37°C, 5% CO_2_, and 95% humidity. Following incubation, the supernatants were removed and assayed for IL-2, IL-10, and IFN-*γ* levels by suspension array (Bio-Rad). Background levels of cytokine production were determined using nonpulsed CD11c-enriched cells cocultured with MLN cells from HSV-1-infected mice treated with anti-PD-L1, anti-PD-L2, or control IgG. Background levels of cytokine production were subtracted from corresponding UV-inactivated HSV-1-pulsed samples.

### 2.7. MLN CD11c Cell Coculture

To assess MLN CD11c^+^ cell function, CD11c^+^ cells were enriched from the MLN of HSV-1-infected mice treated with anti-PD-L1, anti-PD-L2, or control IgG using MACS beads and column (Miltenyi Biotec). CD11c-enriched MLN cells (6.5 × 10^4^ cells/well) were seeded into a 96-well round-bottom plate and treated with UV-inactivated HSV-1 at an MOI of 5 for 2 hrs. Following incubation, the media was removed from the MLN cells, and 1 × 10^6^ spleen cells from HSV gB-specific T-cell receptor transgenic mice were added. Cultures were incubated for 3 days at 37°C, 5% CO_2_, and 95% humidity. Following incubation, the supernatants were removed and assayed for IL-2, IL-10, and IFN-*γ* levels by suspension array (Bio-Rad). Background levels of cytokine production were determined using nonpulsed CD11c-enriched cells from HSV-1-infected mice treated with anti-PD-L1, anti-PD-L2, or control IgG, cocultured with MLN cells from uninfected C57BL/6 mice. Background levels of cytokine production were subtracted from corresponding UV-inactivated HSV-1 pulsed samples. Average background levels were 9.3 pg/mL for IL-2, 3274 pg/mL for IL-10, and 347.8 pg/mL for IFN-*γ*.

### 2.8. Statistics

The statistical module Prism was used to perform unpaired two-tailed Student's *t*-test and analysis of variance (ANOVA) with Tukey's *t-*test. All error bars represent the standard errors of the means. A *P* value <0.05 was considered significant comparing isotypic control to anti-PD-1-, anti-PD-L1-, or anti-PD-L2-treated mice.

## 3. Results

### 3.1. Anti-PD-L1 Antibody Treatment Increases HSV-1 Viral Load

**Figure 1:**
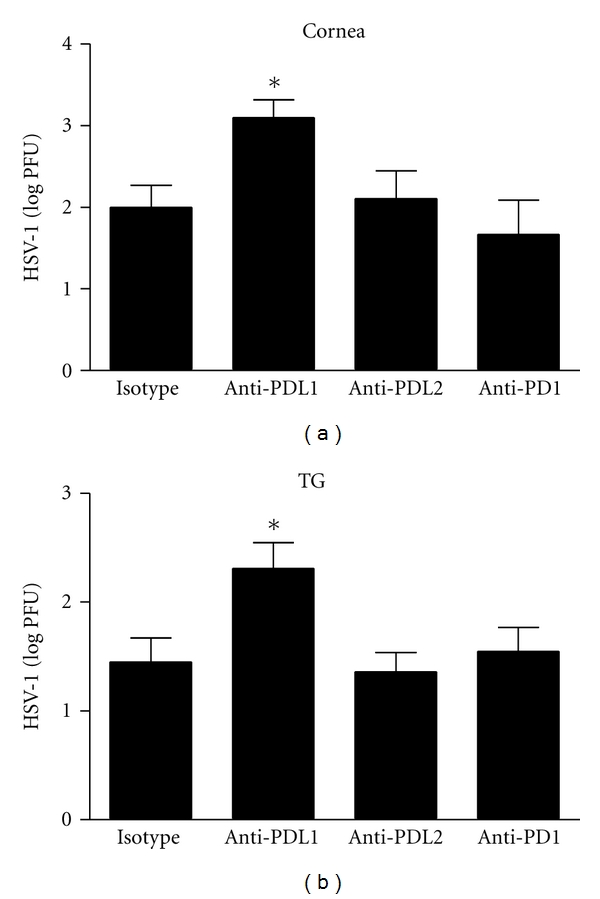
HSV-1 viral load following anti-PD-L1, anti-PD-L2, or anti-PD-1 antibody treatment. C57BL/6 mice were infected with 1,000 PFU HSV-1/cornea. At days 2, 4, and 6 p.i. mice were administered neutralizing antibody to PD-L1, PD-L2, PD-1, or control IgG. The corneas (a) and TG (b) were harvested at 7 days p.i. and HSV-1 viral titers were assessed by plaque assay. This figure summarizes three experiments (*n* = 9). Each bar represents the mean ± SEM. *P < 0.05 comparing the indicated group to the isotypic control antibody-treated group.

To assess the impact of PD-1 : PD-L signaling during HSV-1 infection, C57BL/6 mice were infected with HSV-1 and then treated with anti-PD-L1, anti-PD-L2, anti-PD-1, or isotypic control IgG. At 7 days p.i., cornea and TG were removed and assayed for infectious virus by plaque assay. Viral titers were significantly elevated in the cornea and TG of anti-PD-L1 antibody-treated mice in comparison to anti-PD-L2 or isotypic control-treated mice (Figure 1). By comparison, there was no difference in the level of HSV-1 recovered in the cornea and TG of anti-PD-1 antibody-treated mice compared to control-treated or anti-PD-L2-treated animals (Figure 1).

### 3.2. Impact of PD-L1 and PD-L2 Neutralizing Antibody Treatment on MLN Leukocyte Subpopulations

**Figure 2:**
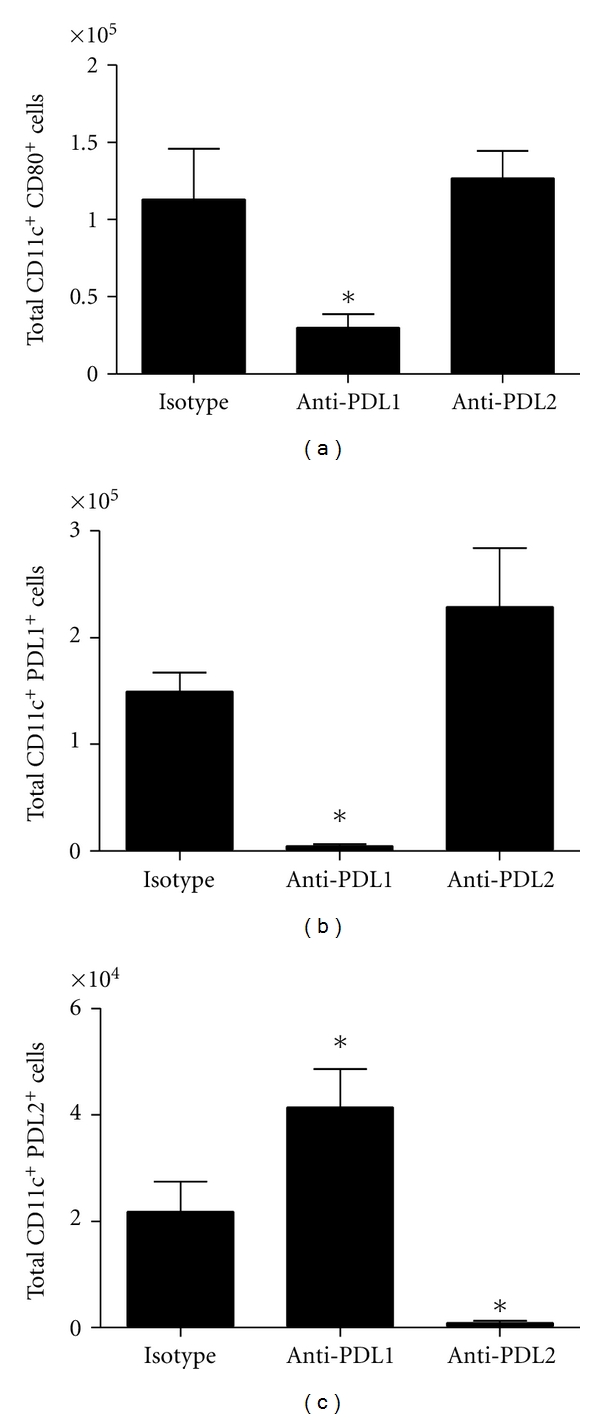
MLN CD11c^+^ leukocyte subpopulations following HSV-1 infection and treatment with anti-PD-L1 or anti-PD-L2 antibody. C57BL/6 mice were infected with 1,000 PFU HSV-1/cornea. At days 2, 4, and 6 p.i. mice were administered neutralizing antibody to PD-L1, PD-L2, or control IgG. At day 7 p.i. mice were exsanguinated and the MLN was processed for flow cytometry. (a) Total CD11c^+^ CD80^+^ cells. (b) Total CD11c^+^ PD-L1^+^ cells. (c) Total CD11c^+^ PD-L2^+^ cells. This figure summarizes three experiments (*n* = 9). Each bar represents the mean ± SEM. *P < 0.05 comparing the indicated group to the isotypic control antibody-treated group.

To elucidate the mechanism by which administering neutralizing PD-L1 antibody increases HSV-1 viral titers, we initially investigated possible changes in the leukocyte subpopulations of the MLN from HSV-1-infected, anti-PD-L1-, anti-PD-L2-, or control IgG-treated mice using flow cytometry. As expected but never before reported, treatment of infected mice with PD-L1 neutralizing antibody significantly decreased the PD-L1-expressing CD11c^+^ cells in the MLN (Figure 2(b)) and significantly increased the number of PD-L2-expressing CD11c^+^ dendritic cells (Figure 2(c)). Animals treated with PD-L2 neutralizing antibody were found to possess significantly fewer PD-L2-expressing CD11c^+^ dendritic cells (Figure 2(c)) in the MLN but with no significant change in the number of PD-L1-expressing dendritic cells (Figure 2(b)). Of interest, there was a noted loss of CD11c^+^ cells expressing CD80^+^ in the mice treated with anti-PD-L1 but not anti-PD-L2 antibody or isotypic control (Figure 2(a)). However, there was no difference in the number of CD11c^+^ dendritic cells expressing CD80 in the MLN of anti-PD-L2 antibody-treated mice in comparison to control-treated mice (Figure 2(a)). Whereas CD86 is a costimulatory molecule that is also expressed by activated dendritic cells, the number of MLN CD11c^+^ cells that express CD86 in response to HSV-1 is low and therefore, the measurement of CD86 expression was not pursued.

**Figure 3:**
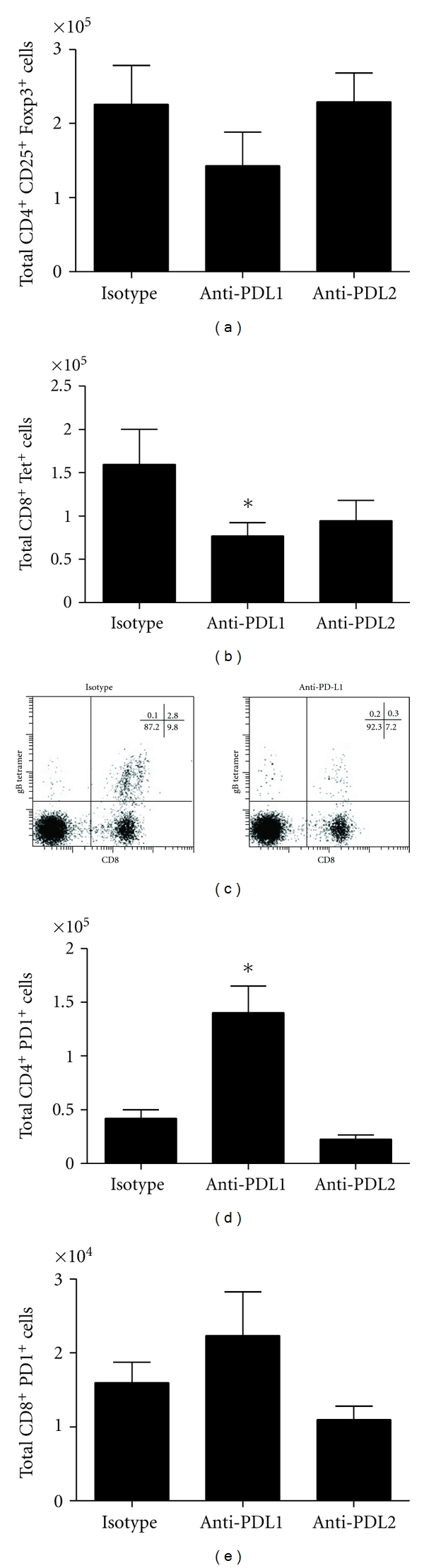
MLN T-cell subpopulations following HSV-1 infection and treatment with anti-PD-L1 or anti-PD-L2 antibody. C57BL/6 mice were infected with 1,000 PFU HSV-1/cornea. At days 2, 4, and 6 p.i. mice were administered neutralizing antibody to PD-L1, PD-L2, or control IgG. At day 7 p.i. mice were exsanguinated and the MLN was processed for flow cytometry. (a) Total CD4^+^ CD25^+^ Foxp3^+^ cells. (b) Total CD8^+^ Tet^+^ cells. (c) Representative flow diagrams of lymph node stained for CD8 and expression of the TCR specific for the HSV-1 gB-derived epitope SSIEFARL, as indicated by staining with tetramer. (d) Total CD4^+^ PD-1^+^cells. (e) Total CD8^+^ PD-1^+^ cells. This figure summarizes three experiments (*n* = 9). Each bar represents the mean ± SEM. *P < 0.05 comparing the indicated group to the isotypic control antibody-treated group.

Since there was a loss of the primary costimulatory molecules that drive clonal expansion of T cells, we next investigated the effector immune cells associated with viral surveillance. Treatment of HSV-1-infected mice with anti-PD-L1 or anti-PD-L2 antibody did not modify expansion of the CD4^+^ or CD8^+^ T-cell populations residing in the MLN (data not shown). Moreover, there was no change in the number of CD4^+^  T_reg_ (CD3^+^CD4^+^Foxp3^+^) cells in the MLN comparing control-treated to anti-PD-L1 and anti-PD-L2 antibody-treated mice (Figure 3(a)). However, there was a significant reduction in the HSV-1 gB-specific (Tet^+^), CD8^+^ T-cell population (Figures 3(b) and 3(c)). Anti-PD-L2 antibody treatment also suppressed the number of HSV-1 gB-specific (Tet^+^), CD8^+^ T-cell population but the loss was not significant (Figures 3(b) and 3(c)). It should also be noted treatment of HSV-1 infected mice with anti-PD-L1 antibody significantly increased the number of CD4^+^ PD1^+^ T cells within the MLN but had no impact on PD1^+^ expression on CD8^+^ T cells (Figures 3(d) and 3(e)).

### 3.3. Leukocyte Profile of TG and Cornea Following Anti-PD-L1 or Anti-PD-L2 Treatment

Since anti-PD-L1 antibody-treated, HSV-1-infected mice displayed selective changes in leukocyte subpopulations in the draining lymph node and possessed significantly more virus in the cornea and TG following ocular infection, we evaluated the leukocyte subpopulations with the infected tissue as a means to associate viral load with change in leukocyte infiltration. Analysis of TG leukocyte subpopulations including NK cells (CD3^−^ NK1.1^+^), CD4 T cells (CD3^+^ CD4^+^), CD8 T cells (CD3^+^ CD8^+^), HSV-1 gB-specific CD8 T cells (Tet^+^ CD8^+^), plasmacytoid dendritic cells (CD11c^+^ B220^+^), conventional dendritic cells (CD11c^+^ B220^−^), activated macrophages (F4/80^+^ Gr1^+^), and neutrophils (F4/80^−^ Gr1^+^) revealed no significant differences (data not shown). Similar to the above results, analysis of infiltrating CD4 T cells (CD3^+^ CD4^+^), CD8 T cells (CD3^+^ CD8^+^), and HSV-1 gB-specific CD8 T cells (Tet^+^ CD8^+^) into the cornea showed no significant differences among the different treatment groups (data not shown). The number of CD4^+^ PD1^+^ T cells was also determined for the cornea of HSV-1 infected mice and no significant differences were observed among the different treatment groups (data not shown).

### 3.4. Cytokine Levels of the TG and Cornea Following Anti-PD-L1 or Anti-PD-L2 Treatment

**Figure 4:**
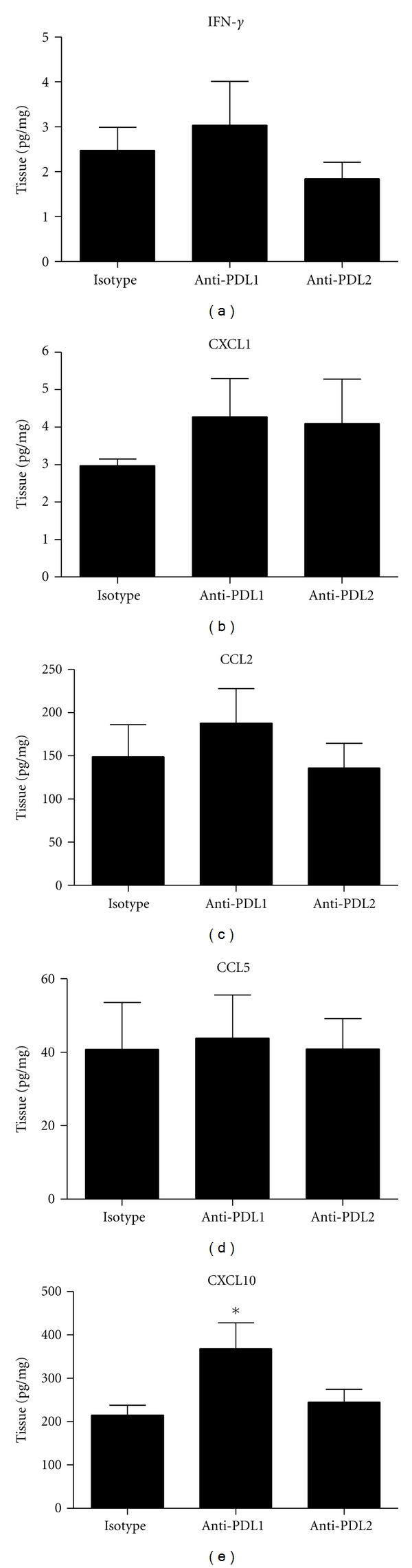
Cytokine expression following HSV-1 infection and treatment with anti-PD-L1 or anti-PD-L2 antibody. C57BL/6 mice were infected with 1,000 PFU HSV-1/cornea. At days 2, 4, and 6 p.i. mice were administered neutralizing antibody to PD-L1, PD-L2, or control IgG. At day 7 p.i. mice were exsanguinated and the TG was processed for suspension array or ELISA. (a) IFN-*γ* levels. (b) CXCL1 levels. (c) CCL2 levels. (d) CCL5 levels. (e) CXCL10 levels. Cytokine levels are a summary of two to three experiments (*n* = 6–9). Each bar represents the mean ± SEM expressed in pg/mg of tissue. *P < 0.05 comparing the indicated group to the isotypic control antibody-treated group.

Cytokines and chemokines at the site of infection dramatically impact both the innate and adaptive immune response. Regarding ocular HSV-1 infection, IFN-*γ*, CCL2, CCL5, CXCL1, and CXCL10 have been found to be expressed in the cornea and TG and are thought to be instrumental in the clearance of the pathogen directly or indirectly through the recruitment of effector immune cells [21]. Therefore, we investigated these analytes in the cornea and TG as another means to explain differences in viral load between the anti-PD-L1 antibody-treated mice to the control and anti-PD-L2 antibody-treated groups. Levels of the antiviral cytokine IFN-*γ* were not found to be different in the TG comparing all groups of treated animals (Figure 4(a)). In a similar display, the chemokines CCL2, CCL5, and CXCL1 levels were also found to be consistently expressed in the TG of the various treated mouse groups (Figure 4(b)–4(d)). However, CXCL10 was significantly elevated in the TG of anti-PD-L1-treated mice (Figure 4(e)). Comparison of IFN-*γ*, CCL2, CCL5, CXCL1, and CXCL10 levels from the cornea of different antibody treatment groups revealed no differences (data not shown).

### 3.5. Functional Capabilities of MLN T Cells and Dendritic Cells Following Blockade of PD-1 : PD-L Signaling

**Figure 5:**
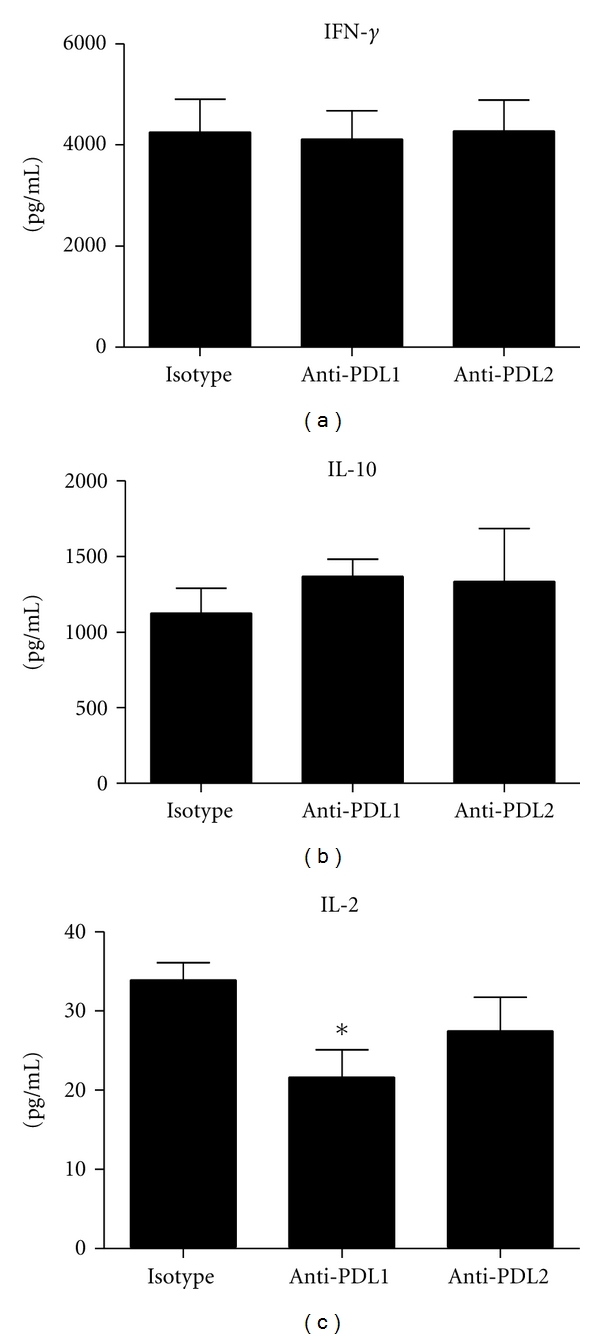
Functional analysis of MLN CD11c^+^ cells following HSV-1 infection and treatment with anti-PD-L1 or anti-PD-L2 antibody. C57BL/6 mice were infected with 1,000 PFU HSV-1/cornea. At days 2, 4, and 6 p.i. mice were administered neutralizing antibody to PD-L1, PD-L2, or control IgG. At day 7 p.i. the mice were exsanguinated and CD11c^+^ cells were highly enriched from the MLN. CD11c-enriched MLN cells were pulsed with UV-inactivated HSV-1 and cocultured with HSV gB-specific T-cell receptor T cells of transgenic mice. Following incubation, the supernatants were removed and assayed for IL-2 (a), IL-10 (b), and IFN-*γ* (c) levels by suspension array. Background cytokine levels were determined using nonpulsed CD11c^+^-enriched MLN cells. Background levels of cytokine production were subtracted from corresponding UV-inactivated HSV-1-pulsed samples. This figure summarizes two experiments (*n* = 6). Each bar represents the mean ± SEM. *P < 0.05 comparing the indicated group to the isotypic control antibody-treated group.

Since the infiltration of leukocytes into infected tissue may not reflect the status of the cells in terms of antigen response, the functional capabilities of T cells from HSV-1-infected mice treated with anti-PD-L1, anti-PD-L2, or isotype control IgG were assessed. Due to the low number of T cells residing in the cornea and TG, it was not possible to accurately measure their status on a per animal basis. Consequently, MLN were isolated from infected mice and processed into single-cell suspension. Lymph node cells from isotypic control, anti-PD-L1, or anti-PD-L2-antibody treated mice were then cocultured with CD11c-enriched cells from untreated, non-infected mouse spleens. The CD11c-enriched cells were pulsed with UV-inactivated HSV-1 prior to culturing. Following 6 days of incubation, the supernatants from cultured cells were collected and assayed for select cytokine content by suspension array. The results show the MLN T cells from antibody-treated groups of mice responded similarly to antigen-pulsed dendritic cells in the production of IL-2, IL-10, and IFN-*γ* (data not shown). Since the data suggested a deficiency was not found at the level of the T cell, the efficiency of antigen presentation was investigated. CD11c-enriched MLN cells from HSV-1 infected mice treated with anti-PD-L1, anti-PD-L2, or isotype control IgG were pulsed with UV-inactivated HSV-1 and cocultured with spleen cells from naive HSV-1 gB-specific T-cell receptor transgenic mice. Following 3 days of incubation, supernatants were collected and assayed for cytokine content. As seen in Figure 5(c), there was a significant decrease in IL-2 production by the HSV gB-specific T cells. Likewise, the levels of IL-2 from the HSV gB-specific T cells were also reduced when incubated with peptide-pulsed CD11c-enriched dendritic cells from the anti-PD-L2 antibody-treated mice but such levels did not reach significance (Figure 5(c)). In contrast, nearly equivalent levels of IL-10 and IFN-*γ* were found in the supernatants of HSV gB-specific T cells stimulated with peptide pulsed dendritic cells from all treatment groups of mice (Figures 5(a) and 5(b)). It was somewhat surprising such a robust response was found in the HSV gB-specific transgenic T cells relative to IFN-*γ* and IL-10 production, two cytokines that typically drive opposing T_H_ responses.

## 4. Discussion

 In this study, we report the administration of PD-L1 antibody during acute HSV-1 infection significantly increases viral load in the cornea and TG associated with a loss of PD-L1-expressing dendritic cells and attenuated capacity to present antigen by CD11c^+^ cells. During chronic LCMV infection, treatment of mice with antibody that blocks PD-1 : PD-L1 signaling was found to improve the effector function of CD8^+^ T cells and decrease viral titers [22]. The therapeutic effects of blocking the PD-1 pathway via anti-PD-L1 antibody treatment is further exemplified during HIV infections, wherein antibody treatment enhanced HIV-specific CTL proliferation and cytokine production [23]. It is likely the prevention of PD-1 : PD-L1 interaction attenuates T-cell exhaustion and facilitates CD8^+^ T-cell recovery as demonstrated in chronic LCMV infection [24]. However, there are various levels by which PD-L1 expression may influence the adaptive immune response.

An increase in T_reg_ cells could impair effector T cells and, as a consequence, result in the augmentation in viral load. However, anti-PD-L1 treatment during acute HSV-1 infection did not modify the number of T_reg_ cells found in the MLN. Nor did anti-PD-L1 antibody administration alter the expansion of T-cell populations or NK cells residing in the MLN. However, the costimulatory molecule, CD80, was significantly downregulated on CD11c^+^ cells from mice treated with neutralizing PD-L1 antibody. The effect was specific since an equivalent dosage and treatment regimen of anti-PD-L2 antibody had no such effect on CD11c^+^ cells within the MLN. The loss of CD80 expression correlated with a significant reduction in the number of HSV-1 gB-specific CD8^+^ T cells within the MLN. A recent study has indicated that depletion of dendritic cells or blockade of CD80/86 signaling results in decreased virus-specific CD8^+^ T cells and impaired viral clearance during influenza infection [25]. It was also suggested that the absence of CD28 signaling via CD80/CD86 could inhibit autocrine IL-2 secretion by T cells. Furthermore, recombinant IL-2 treatment of CD80^−/−^CD86^−/−^ mice restored the expansion of effector CD8 T cells [25]. The above results suggest neutralizing PD-L1 may disrupt proper antigen presentation through the down regulation of costimulatory molecules such as CD80/CD86.

Whereas we found a reduction in the number of antigen-specific cytotoxic T cells in the draining lymph node of anti-PD-L1 antibody-treated mice, such a loss was not translated within the infected tissue as similar numbers of HSV-1 gB-specific CD8 T cells were recovered from the TG of all HSV-1-infected, antibody-treated groups. Therefore, it was hypothesized the function of the effector cells rather than absolute number of cells may be compromised and thus, unable to efficiently monitor virus replication and spread. Due to the low number of cells recovered from the infected tissue, we investigated T cells obtained from the draining lymph nodes, the MLN. Since PD-1 expression was enhanced on CD4^+^ T cells residing in the MLN of anti-PD-L1 antibody-treated mice, and PD-1 expression has previously been associated with suppression of antiviral immunity [26], the predicted inherent deficiency in effector function appeared to be a legitimate target of investigation. Using a system of *in vitro* coculturing, we were able to assess MLN T-cell function from infected, antibody-treated mice. The analysis revealed T cells from HSV-1 infected, anti-PD-L1 or anti-PD-L2 antibody-treated mice were able to produce IL-2, IL-10, and IFN-*γ* similar to T cells from the isotype antibody control-treated mice. This result was in contrast to that observed by Jun et al., wherein MLN T cells isolated from anti-PD-L1 antibody-treated mice pulsed with UV-inactivated HSV-1 produced twice as much IFN-*γ* as cells from the control mice [15]. Differences between the previous study [15] and current study include the use of HSV-resistant C57BL/6 mice (for the current study) as compared to BALB/c, the strain of virus (McKrae versus RE) used to infect mice, and the course of anti-PD-L1 antibody treatment (days 2, 4 and 6 p.i. in the present study versus the day before and 2 days p.i. in the previous study). Moreover, our study validated the loss of PD-L1 expressing dendritic cells, whereas the study by Jun et al. did not. In a reciprocal fashion, we found CD11c-enriched cells from the MLN of anti-PD-L1 antibody-treated mice were deficient in their ability to stimulate HSV gB-specific CD8^+^ T-cell production of IL-2. Such an observation is consistent with the reduction in clonal expansion of the antigen-specific CD8^+^ T cells in the MLN of the anti-PD-L1 antibody-treated mice. However, the results are not consistent with a global functional loss in antigen presentation since copious amounts of IFN-*γ* and IL-10 were produced by the HSV gB-specific T cells in response to HSV-pulsed APCs. IL-2 levels have more recently been reported to complement or enhance immune responses including antiviral activity [27]. However, how changes in IL-2 production may facilitate viral clearance in the presence study is currently unclear.

Recent studies involving HSV-1 ocular infection and PD-L1 have focused on herpetic stromal keratitis (HSK), the expression of PD-L1, and the impact on latency [15–17, 25, 26]. Initial studies revealed PD-1 expression was upregulated on CD4^+^ T cells in the draining LN following HSV-1 infection and furthermore, *in vivo* blockade of PD-1 : PD-L1 signaling enhanced HSK [15]. More recent studies have focused on T-cell exhaustion and HSV-1 latency. Studies by Frank et al. revealed that CD4^+^ T-cell ablation during acute HSV-1 infection alters the maintenance of HSV-1 latency and results in a population of HSV-1-specific CD8^+^ T cells which are functionally compromised [28]. This T-cell population also exhibited increased levels of PD-1 expression [28]. However, blockade of PD-1 : PD-L1 signaling following CD4^+^ T-cell ablation restored the functional capabilities of the HSV-1-specific CD8^+^ T cells [28]. Further studies investigating HSV-1 latency revealed a strong correlation between the severity of eye disease, latency, and the expression of PD-1 in the TG of infected mice [16, 17, 29]. More recently, a direct role for HSV-1 LAT (latancy-associated transcript) has been described wherein LAT expression upregulates PD-L1 expression on neuronal- derived cells, further promoting an environment for T-cell exhaustion [30]. Together, these studies suggest PD-1 : PD-L1 signaling mediates T-cell exhaustion and latency during HSV-1 infection. However, no study until now has investigated the impact of PD-1 : PD-L1 signaling during acute HSV-1 infection. Addition of this data to the current knowledge base regarding PD-1 : PD-L1 signaling and chronic viral infections further highlights the multifaceted nature of this signaling pathway.
